# Supplementary material for: Hepatitis Delta Virus Reporting Requirements in the United States and Territories: A Systematic Review
Source: Open Forum Infect Dis. 2024 Feb 8;11(4):ofae076. doi: 10.1093/ofid/ofae076 (PMC11000145; doi:10.1093/ofid/ofae076)
Supplement: ofae076_Supplementary_Data [file ofae076_supplementary_data.zip › Supplemental_Table_1.pdf]

Supplemental Table 1. United States and territories requiring reporting of HDV cases.

State or Territory	Health Department
Arizona (AZ)	Arizona Department of Health Services
California (CA)	California Department of PublicHealth
Colorado (CO)*	Colorado Department of Public Health and Environment
Delaware (DE)*	Delaware Division of Public Health
Florida (FL)	Florida Health
Georgia (GA)	GNR Public Health
Guam (GU)	Guam Department of Public Health and Social Services
Illinois (IL)	Illinois Department of Public Health
Indiana (IN)	Indiana State Department of Health
Iowa (IA)	IDPH Iowa Deparment of Public Health
Kansas (KS)	Kansas Department of Health and Environment
Louisiana (LA)	Louisiana Health department
Maine (ME)	Division of the Maine Department of Health and Human Services
Maryland (MD)	Maryland Department of Health
Massachusetts (MA)	Executive Office of Health and Human Services
Minnesota (MN)	Minnesota Department of Health
Nebraska (NE)	Nebraska Department of Health and Human Resources
Nevada (NV)	Nevada department of Health and Human Sevices
North Dakota (ND)	North Dakota Health Department
Ohio (OH)	Ohio Department of Health
Oregon (OR)	Oregon Health Authority
Pennsylvania (PA)	Pennsylvania Department of Health
Puerto Rico (PR)*	Puerto Rico Department of Health
Rhode Island (RI)	State of Rhode Island Department of Health
South Carolina (SC)	South Carolina Health Department
Utah (UT)	Utah Health Department
Virginia (VA)*	Virginia Department of Health
Washington (WA)	Washington State of Health Department
West Virginia (WV)	West Virginia Department of Health and Human Resources
Wisconsin (WI)	Wisconsin Department of Health Services
Wyoming (WY)	Wyoming Department of Health

* Health Departments note required reporting of other viral hepatitis
