# Supplementary material for: Hepatitis Delta Virus Reporting Requirements in the United States and Territories: A Systematic Review
Source: Open Forum Infect Dis. 2024 Feb 8;11(4):ofae076. doi: 10.1093/ofid/ofae076 (PMC11000145; doi:10.1093/ofid/ofae076)
Supplement: ofae076_Supplementary_Data [file ofae076_supplementary_data.zip › Supplemental_Table_2.pdf]

Supplemental Table 2. United States and territories that do not require reporting of HDV cases.

State or Territory	Health Department Source Hyperlinks:
Alabama (AL)	Alabama Public Health
Alaska (AK)	Alaska department of Health
American Samoa	American Samoa Department of Commerce
Arkansas (AR)	Arkansas Department Of Health
Connecticut (CT)	Connecticut State Department of Public Health
District of Columbia (DC)	District of Columbia Department of Health
Hawaii (HI)	State of Hawaii, Department of Health
Idaho (ID)	Idaho Department Health and Welfare
Kentucky (D)	Kentucky Cabinet for Health and Family Services
Michigan (MI)	Michigan Department of Health and Human Services
Mississippi (MS)	Mississippi State Department of Health
Missouri (MS)	Missouri Department of Health and Senior Services
Montana (MT)	Montana Department of Public Health and Human Services
New Hampshire (NH)	New Hampshire Department of Health and Human Services
New Jersey (NJ)	State of New Jersey Department of Health
New Mexico (NM)	New Mexico Department of Health
New York (NY)	New York Department of Health
North Carolina (NC)	North Carolina Department of Health and Human Services
Northern Mariana Island (MP)	CNMI Department of Public Health
Oklahoma (OK)	Oklahoma State Department of Health
South Dakota (SD)	South Dakota Department of Health
Tennessee (TN)	Tennessee Department of Health
Texas (TX)	Texas Health and Human Services
Vermont (VT)	Vermont Health Deparment
Virgin Islands (VI)	Virgin Islands Department of Health
